# Supplementary material for: A fault-intrusion-tolerant system and deadline-aware algorithm for scheduling scientific workflow in the cloud
Source: PeerJ Comput Sci. 2021 Nov 2;7:e747. doi: 10.7717/peerj-cs.747 (PMC8576554; doi:10.7717/peerj-cs.747)
Supplement: Supplemental Information 6 [file peerj-cs-07-747-s006.zip › Myproject1.1/docs/index-files/index-22.html]

W-Index


JavaScript is disabled on your browser.


- Overview
- Package
- Class
- Use
- Tree
- Deprecated
- Index
- Help

- Prev Letter
- Next Letter

- Frames
- No Frames

- All Classes

A B C D E F G H I J L M N O P Q R S T U V W Z 


## W

wait(int, Predicate) - Static method in class org.cloudbus.cloudsim.core.CloudSim
:   Sets an entity's state to be waiting.

WAIT\_RECV - Static variable in class org.cloudbus.cloudsim.network.datacenter.NetworkConstants


WAIT\_SEND - Static variable in class org.cloudbus.cloudsim.network.datacenter.NetworkConstants


waitForEvent(Predicate) - Method in class org.cloudbus.cloudsim.core.SimEntity
:   Wait for an event matching a specific predicate.

waiting(int, Predicate) - Static method in class org.cloudbus.cloudsim.core.CloudSim
:   Checks if events for a specific entity are present in the deferred event queue.

WAITING - Static variable in class org.cloudbus.cloudsim.core.SimEntity
:   The Constant WAITING.

WeibullDistr - Class in org.cloudbus.cloudsim.distributions
:   The Class WeibullDistr.

WeibullDistr(Random, double, double) - Constructor for class org.cloudbus.cloudsim.distributions.WeibullDistr
:   Instantiates a new weibull distr.

WeibullDistr(double, double) - Constructor for class org.cloudbus.cloudsim.distributions.WeibullDistr
:   Instantiates a new weibull distr.

WorkflowApp - Class in org.cloudbus.cloudsim.network.datacenter
:   WorkflowApp is an example of AppCloudlet having three communicating tasks.

WorkflowApp(int, int, double, int, int) - Constructor for class org.cloudbus.cloudsim.network.datacenter.WorkflowApp


WorkflowEngine - Class in org.workflowsim
:   WorkflowEngine represents a engine acting on behalf of a user.

WorkflowEngine(String) - Constructor for class org.workflowsim.WorkflowEngine
:   Created a new WorkflowEngine object.

WorkflowEngine(String, int) - Constructor for class org.workflowsim.WorkflowEngine


workflowId - Variable in class org.workflowsim.failure.FailureRecord
:   the workflow id (user id in this version).

WorkflowParser - Class in org.workflowsim
:   WorkflowParser parse a DAX into tasks so that WorkflowSim can manage them

WorkflowParser(int) - Constructor for class org.workflowsim.WorkflowParser
:   Initialize a WorkflowParser

WorkflowParser(int, String, String, String) - Constructor for class org.workflowsim.WorkflowParser


WorkflowPlanner - Class in org.workflowsim
:   WorkflowPlanner supports dynamic planning.

WorkflowPlanner(String) - Constructor for class org.workflowsim.WorkflowPlanner
:   Created a new WorkflowPlanner object.

WorkflowPlanner(String, int) - Constructor for class org.workflowsim.WorkflowPlanner


WorkflowScheduler - Class in org.workflowsim
:   WorkflowScheduler represents a scheduler acting on behalf of a user.

WorkflowScheduler(String) - Constructor for class org.workflowsim.WorkflowScheduler
:   Created a new WorkflowScheduler object.

WorkflowSimExample1 - Class in org.workflowsim.examples
:   This WorkflowSimExample creates a workflow planner, a workflow engine, and
    one schedulers, one data centers and 20 vms.

WorkflowSimExample1() - Constructor for class org.workflowsim.examples.WorkflowSimExample1


WorkflowSimExample2 - Class in org.workflowsim.examples
:   This WorkflowSimExample creates a workflow planner, a workflow engine, and
    two schedulers, two data centers and 20 vms.

WorkflowSimExample2() - Constructor for class org.workflowsim.examples.WorkflowSimExample2


WorkflowSimTags - Class in org.workflowsim
:   This WorkflowSimTags include tags that are not supported in CloudSimTags

WorkloadFileReader - Class in org.cloudbus.cloudsim.util
:   This class is responsible for reading resource traces from a file and creating a list of jobs.

WorkloadFileReader(String, int) - Constructor for class org.cloudbus.cloudsim.util.WorkloadFileReader
:   Create a new `WorkloadFileReader` object.

WorkloadModel - Interface in org.cloudbus.cloudsim.util
:   This interface defines what a workload model should provide.

write(String) - Method in class org.cloudbus.cloudsim.Cloudlet
:   Writes this particular history transaction of this Cloudlet into a log.

writeDataColumn(List<? extends Number>, String) - Static method in class org.cloudbus.cloudsim.examples.power.Helper
:   Write data column.

writeDataRow(String, String) - Static method in class org.cloudbus.cloudsim.examples.power.Helper
:   Write data row.

writeMetricHistory(List<? extends Host>, PowerVmAllocationPolicyMigrationAbstract, String) - Static method in class org.cloudbus.cloudsim.examples.power.Helper
:   Write metric history.

A B C D E F G H I J L M N O P Q R S T U V W Z

- Overview
- Package
- Class
- Use
- Tree
- Deprecated
- Index
- Help

- Prev Letter
- Next Letter

- Frames
- No Frames

- All Classes
